# New York State Institute for the Study of Malignant Disease

**Published:** 1911-06

**Authors:** 


### BUFFALO MEDICAL JOURNAL

Vol. LXVI. JUNE, 1911. No. 11

### ORIGINAL COMMUNICATIONS

### New York State Institute for the Study of Malignant Disease

BY affixing his signature to the bill introduced in the legislature of the state of New York appropriating $65,000 for the erection and equipment of a hospital for the treatment of malignant diseases in connection with the Gratwick Laboratory Governor Dix made it possible for Buffalo to take that place as a center of scientific research to which the excellent work done at the laboratory during the past decade entitles it.

That a comprehensive knowledge of the provisions of the law may be had the bill creating the hospital is here printed in full:

AN ACT.

To amend the public health law, in relation to the establishment of a state institute for the study of malignant disease at Buffalo, providing for its management and control, and making an appropriation therefor.

*The People of the State of New York, represented in Senate and Assembly, do enact as follows:*

Section 1. Chapter forty-nine of the laws of nineteen hundred and nine, entitled "An act in relation to the public health, constituting chapter forty-five of the consolidated laws," is hereby amended by adding thereto, after article seventeen thereof, a new article eighteen in place of the present article eighteen hereinafter renumbered nineteen, to read as follows:

ARTICLE 18.

STATE INSTITUTE FOR THE STUDY OF MALIGNANT DISEASE.

Section 344. Establishment of institute.
Section 345. Management and control; board of trustees.
Section 346. Objects and purposes of the institute.
Section 347. Director of the institute.

Section 344. Establishment of institute. There shall be established in the city of Buffalo an institute, to be known as the state institute for the study of malignant disease, for the objects and purposes hereinafter set forth.

Section 345. Management and control; board of trustees. The general management and control of said institute shall be vested in a board of trustees consisting of seven members, one of whom shall be the state commissioner of health, ex officio.

The remaining members shall, as often, as the positions of the several original members constituted by the act of which this article is a part, become vacant, be appointed by and may be removed at the pleasure of the governor. Said trustees shall serve without compensation, and said board shall meet quarterly and shall hold an annual meeting in November to receive the annual report of the director and to prepare for transmission to the legislature its report upon the work of the preceding year. The board shall audit the annual expenses of the institute and appoint the director. The board of trustees shall, within the limits of the annual appropriation made therefor, fix all salaries of officers and employees of the institute and authorise all disbursements. The board may meet any time on the call of the chairman and shall be allowed necessary traveling expenses in attending the fixed meetings or any special meetings. At least two of the trustees shall be residents of Buffalo or vicinity and one of them shall be a member of the medical faculty or of the council of the university of Buffalo.

Section 346. Objects and purposes of the institute. The institute shall conduct investigations into the cause, nature, mortality rate, treatment, prevention and cure of cancer and allied diseases, and may receive in its hospital for study, experimental or other treatment, cases of cancer and allied diseases free of charge. It shall publish from time to time the results of its investigations for the benefit of humanity and shall from time to time collect its publications into the form of a scientific report for distribution to scientific bodies and to medical scientists and qualified members of the medical profession. The direction of research work in whole or in part toward malignant diseases other than cancer shall not be a violation of the conditions of the grants made under the provisions of which this article is a part.

Section 347. Director of the institute. A director of the institute shall be appointed by the board of trustees and shall serve until his successor shall have been duly appointed and qualified. He shall be a trained scientist and shall have sole executive direction of the work of the institute. He shall appoint all members of the staff, subject to the approval of the board of trustees, and he shall appoint and dismiss at will all other employees of the institute.

Section 2. The sum of sixty-five thousand dollars ($65,000), or so much thereof as may be needed, is hereby appropriated out of any money in the treasury not otherwise appropriated, for the purpose of constructing and equipping a suitable building for a state hospital for research purposes, as hereinafter particularly described, upon ground adjoining the Gratwick laboratory at Buffalo, New York; such hospital and the Gratwick laboratory building shall be used by and for the state institute for the study of malignant disease, established by article eighteen of the public health law, as added by this act, but the laboratory building shall continue to be known as the Gratwick laboratory. Before any part of such appropriation shall be or become available, the land on which the hospital building provided for in this act is to stand, consisting of the plot adjoining the Gratwick laboratory on the west, extending about one hundred and fifty feet to the corner of Oak street, with a depth of about one hundred and seventy-nine feet, shall be conveyed to the people of the state by the owners thereof, and the conveyance approved as to form and manner of execution by the attorney general; and the land upon which the Gratwick laboratory stands, together with the building thereon, and all of its appointments and equipment (heretofore for the purpose of medical research provided for the use of the state by Mrs. William H. Gratwick of Buffalo, New York), shall be by like deed, similarly approved, conveyed to the people of the state. The deeds may contain a condition that the grant is made subject to the agreement on the part of the state that there shall be thereafter maintained thereon an institute for the study of malignant and allied diseases, according to the provisions of this act and that upon the determination by the legislature that such institute shall be no longer maintained at the expense of the state, all of such land with all buildings and improvements thereon and equipment therein shall revert to the university of Buffalo, its successors or assigns, but in that case such reversion to the university of Buffalo shall not include the hospital building except upon the payment by the university of Buffalo to the state of the then duly appraised value of the hospital building.

Section 3. The original members, other than the state commissioner of health, of the board of trustees of the state institute for the study of malignant disease, as established by section three hundred and forty-four of the public health law, as added by this act, shall be the following persons: Roswell Park, M. D., Buffalo, who shall be chairman; William H. Gratwick, Buffalo; Charles Cary, M. D., Buffalo; John G. Milburn, New York; Frederick C. Stevens, Attica, New York; Charles S. Fairchild, New York.

Section 4. The amount hereby appropriated shall be paid by the treasurer upon the warrant of the comptroller upon vouchers approved as hereinafter provided. The plans and specifications for the building herein provided for shall be prepared or approved by the state architect and he may, with the approval of the board of trustees of the institute, employ an architect or architects to prepare the plans and specifications and to locally supervise the work of construction herein provided. All plans and specifications shall be subject to the approval of the director of said institute, appointed as provided by section three hundred and forty-seven of the public health law, as added by this act, and of its board of trustees. The work under this act shall be done by contract except work which in the opinion of the comptroller and the state architect can be done in whole or in part more advantageously by the employment of labor and the purchase of material in the open market. All expenditures under this act shall be made in pursuance of the estimates or pursuant to contracts the form of which shall be prescribed by the state architect. The estimates shall be made to the comptroller in the usual form by the board of trustees of said institute. Where the work estimated for is upon drawings and specifications of the state architect, the estimates shall be subject to his approval also. No item of such appropriation shall be available except for necessary advertising and the preparation of plans until a contract or contracts for the completion of the structure authorised to be erected within the appropriation shall be made. All contracts greater in amount than one thousand dollars shall have the performance thereof secured by a sufficient bond or bonds to be approved by and filed with the comptroller. In the case of any work which shall amount to less than one thousand dollars covered by contract, no surety bond shall be required, provided payment is to be made only after the work has been satisfactorily completed. All payments on contracts shall be made on the certificate of the state architect and the voucher of the board of trustees of said institute after audit of the state comptroller.

Section 5. Present article eighteen of such chapter is hereby renumbered article nineteen thereof.

Section 6. This act shall take effect immediately.

The law is sufficiently broad to include the study of all diseases of malignant character and of unknown origin. In the beginning, in all probability, most of the work done will be confined to the study and treatment of cancer and its allied conditions; but the future holds forth great promise of results in the investigations of such other diseases as Hodgkin's disease, leukemia and pernicious anemia. For the present, however, it is the intention to devote much of the time of the staff of the laboratory to a continuation of the study of cancer in an experimental manner.

It is not by any means intended that the hospital when completed shall open for the indiscriminate reception of cases of malignancy. Only those selected cases which give promise of improvement under experimental study and treatment will be received and then only after careful and thorough study and examination by the staff of the laboratory and hospital.

It is the belief that there is immunity and degrees of immunity and such cases as are admitted will be subject to all the biologic tests in use to determine the exact condition of the patient's resistance. It is fairly definite that there are cases which will show marked improvement provided the natural immunity present be stimulated with one of the vaccines developed through animal experimentation at the Gratwick Laboratory. Those others which have traveled far the pathologic road would in the majority of instances be lessened in resistance by any of the ordinary procedures directed toward stimulation. Thus far it has been impossible to determine with any degree of accuracy in the human just what stage any given case of cancer has reached biologically. One of the by-ways of research which will be explored will be blood conditions and it is hoped through this to be able to state with reasonable degree of certainty just what stage the disease is in and actual blood state which accompanies it.

The immensity of the field which is opened for research work and scientific determination of the malignant conditions can hardly be grasped by the lay mind; and even those of the daily newspapers that gave space to the announcement of the signing of the bill establishing the new hospital and creating the institute saw little in it except the fact that it brought to Buffalo another institution under state control. There was the dollar-wise glance toward financial benefit to the city and the municipal advertising which the establishment of a state fostered department always brings in its wake. There was little or no reference to the invaluable good which will ultimately come to those afflicted with those relentless malignancies covered by the broad term cancer. There was no thought of the serious battle to be waged on scientific lines against those diseases which annually destroy thousands of human lives; no suggestion of the building of a fortification against a dread condition, malignant in its every phase; no praise for the legislators whose votes made possible the attack on those diseases of unknown origin which are the despair of the practising physician and surgeon and in too many instances the death warrant of the one afflicted.

That there may be no misunderstanding concerning the objects and the sphere of the hospital it is well in the beginning, even before it has been erected that the general plan of conduct be made known to the profession insofar as there has been any planning. As this is fundamentally a research institute cases cannot be received indiscriminately as has been stated. Those which are received will be admitted with the distinct understanding that they are to be wholly under the control of the staff and under the treatments outlined by the staff. It will not in any wise interfere with the practice of physicians or the work at existing hospitals, but it is not the idea to saddle the state with the care of the indigent cancer sick.

The importance of the step taken by the state in establishing this institute cannot be overestimated. Being under state control it is unique as the first hospital for purely research work of its kind established in this country and it is destined to become a prominent center of medical research and a large factor in the uplift of medicine and advancement in the study of those disease conditions which come within its scope. The first meeting of the board of trustees will be held before the first of June and organisation effected. The state architect will then prepare plans and specifications for the building and it is expected the hospital will be completed and ready for patients within a year. The capacity as at present planned will be twenty-five beds.

Technical reports of the progress of the work done in the laboratory and hospital will be made from time to time in journals devoted to cancer research and brief resumes as the work of investigation goes forward will be published in the BUFFALO MEDICAL JOURNAL in a department to be devoted to the New York State Institute for the Study of Malignant Disease.

The signing of this bill by Governor Dix marks the culmination of a struggle covering a period of fifteen years to bring about a plan by which serious and consistent study of cancer might be carried on under state aid and a brief history of this effort which happily has now come to success will be of interest at this time.

In 1896 an item appropriating $10,000 was inserted in the legislative supply bill for the establishment at Buffalo of a laboratory for the study of cancer. The then Governor, Frank S. Black, vetoed the appropriation after its passage and wrote a memorandum to the effect that such investigation should be carried on by private means and not with state funds or aid. That killed the project for that year. The following winter Dr. Roswell Park, who initiated the previous effort, enlisted the assistance of Mr. E. H. Butler and the fight for the appropriation was renewed during the session of 1897-98. Mr. Butler invited Governor Black to come to Buffalo and he was the guest of honor at a dinner given at the Ellicott Club by Mr. Butler for the New York State Editors' Association. Dr. Park, Dr. M. D. Mann, Dr. Charles G. Stockton and Dr. Charles Cary met the Governor during his stay in Buffalo and he was informed regarding the great importance for studying cancer and its allied conditions and seeking a method of relief if not absolute cure. Through Mr. Butler's efforts the question was brought before the newspaper editors of the state and there was a general advocacy of the plan. The result was that the supplemental supply bill of that session carried an appropriation of $10,000, the sum originally asked for, and was signed by the Governor. That was the beginning of what has since become the Gratwick laboratory.

It were useless to go into detail concerning the struggles for existence which the laboratory has been compelled to face year after year when appeal and prayer were necessary to secure sufficient funds to carry on a work of broad humanity on scientific lines, while many times the amount asked for was spent by self-bitten statesmen on the strengthening and repair of those bits of financial architecture known as political fences. Fortunately there were friends of influence in the senate and assembly who stood steadfast and shoulder to shoulder with the sponsors of the laboratory in Buffalo, fought the self-centered opposition and won on merit the appropriation until it became an established state charge made necessary by the invaluable work which the laboratory and its workers were doing. While other governments and private interests were spending with lavish hand thousands of dollars in the race for the goal of the discovery of the cause of cancer, Buffalo, with its newly equipped laboratory, took a prominent place and became one of the important centers toward which science turned its eyes with hope for the solution of the problem.

With the announcement of discoveries made in Buffalo the state increased the appropriation and made possible an extension of the work so that now the Gratwick laboratory occupies a place second to none in the world in the importance of its work, and its director Dr. H. R. Gaylord has become one of the world's foremost authorities in the field of cancer research. European scientists learned in research, have visited the laboratory and marveled at its equipment and its completeness. They have seen the methods by which have been produced important results and have expressed wonderment at the accomplishments of this relatively small laboratory whence came discoveries which even the prodigally supported institutions of Europe have not equaled. Twice has the American association for the research of cancer met in Buffalo in recognition of the value of the work being done here.

Under the provisions of the law the state will receive property valued at $85,000, including the handsome laboratory building which was the gift of Mrs. Gratwick; the site on which it stands, which was purchased by friends of the laboratory, and the site for the new hospital building.

The laboratory has been fortunate in its friends; its work has been carried on quietly, modestly and without boastful publicity. Its achievements have been of so important a character as to demand the attention and the praise of scientific bodies and the respect of legislators and laymen who realised without knowing how or why that wonderful strides toward the ultimate solution of the cancer problem were being made by the group of workers in Buffalo. So, when the final effort came, the effort which has given birth to this hospital as an adjunct to a laboratory, thus reversing the usual institutional order, there stood with it a united legislative delegation from Erie county and the bill, introduced in the assembly by Mr. LaRue and in the senate by Mr. Loomis, passed without opposition thus ending fifteen years of struggle for adequate means toward the solution of one of the mysteries of medicine; a struggle which had the consistent assistance of such men as Speaker Wadsworth, senators Hill, Merritt and Stevens, the latter now a member of the board of trustees; the late Governor Higgins and assemblyman Phillips, all unselfish workers in the cause of humanity and faithful friends of the laboratory.

The New York State Institute for the Study of Malignant Disease with its wide scope and its unlimited field of endeavor gives promise of bringing to the state of New York the glory of medical discovery which will revolutionise treatment and be a boon to those most desperately afflicted; and Buffalo will become one of the world's most important centers of scientific research.

